# FGFR1 copy number gain is independently associated with shorter progression-free survival in advanced lung squamous cell carcinoma treated with first-line immune checkpoint inhibitor-based therapy

**DOI:** 10.3389/fimmu.2026.1718660

**Published:** 2026-07-29

**Authors:** Shuya Mu, Xingyuan Li, Yingjia Sun, Yongfeng Yu

**Affiliations:** Department of Medical Oncology, Shanghai Chest Hospital, Shanghai Jiao Tong University School of Medicine, Shanghai, China

**Keywords:** FGFR1, immunotherapy, lung squamous carcinoma, PD-L1, prognosis

## Abstract

**Background:**

Lung squamous cell carcinoma (LUSC) represents a predominant subtype of non-small cell lung cancer (NSCLC) with scarce effective targeted therapeutic options, rendering immune checkpoint inhibitor (ICI)-based regimens the standard first-line treatment for advanced-stage disease. Fibroblast growth factor receptor 1 (FGFR1) copy number (CN) gain is a prevalent genomic aberration in LUSC; nevertheless, the correlation between FGFR1 CN gain and ICI therapeutic efficacy has not yet been clarified. This retrospective single-center study aimed to explore the predictive value of FGFR1 CN gain in advanced LUSC patients receiving first-line ICI monotherapy or combination regimens.

**Methods:**

We retrospectively enrolled 181 patients with stage IIIB–IV advanced LUSC who received first-line ICI-based treatment at Shanghai Chest Hospital from January 2018 to July 2023. Next-generation sequencing (NGS) was performed to detect FGFR1 copy number status, and FGFR1 CN gain was defined as an estimated copy number > 2. Immunohistochemistry was applied to assess PD-L1 expression levels. Progression-free survival (PFS) was set as the primary endpoint, while secondary endpoints encompassed overall survival (OS), objective response rate (ORR), and disease control rate (DCR). Multivariate Cox proportional hazards regression was utilized to identify independent prognostic predictors of PFS.

**Results:**

Of the total 181 participants, 44 patients (24.3%) harbored FGFR1 CN gain. Relative to patients without FGFR1 CN gain, the FGFR1 CN-gain cohort exhibited a significantly lower DCR (84.1% vs. 94.9%, P = 0.015) and shorter median PFS (7.7 months vs. 9.5 months, P = 0.025). No intergroup disparities in ORR or OS reached statistical significance. Multivariate Cox regression further validated that FGFR1 CN gain served as an independent unfavorable prognostic factor for shortened PFS (HR = 1.554, 95% CI: 1.077–2.242, P = 0.018).

**Discussion:**

FGFR1 copy number gain is independently linked to inferior progression-free survival among patients with advanced LUSC undergoing first-line ICI-based treatment. FGFR1 CN gain holds promising potential as a predictive biomarker for suboptimal clinical outcomes in this patient population.

## 1 Introduction

Lung cancer remains the leading cause of cancer death, with high incidence and mortality rates (1). Non-small cell lung cancer (NSCLC) accounts for approximately 85% of all lung cancers, of which lung squamous cell carcinoma (LUSC) constitutes 30%–35%. Notably, around 70% of LUSC patients are diagnosed at locally advanced or metastatic stages, with a 5-year survival rate of less than 10% (2–4). Despite remarkable progress in screening, surgery, radiotherapy, targeted therapy and immunotherapy over the past years, the clinical management of LUSC remains challenging due to its aggressive biological features and highly complex molecular profiles (5–7). The paucity of actionable driver mutations greatly limits the application of precision targeted therapy in this population (8, 9). Accordingly, immune checkpoint inhibitor (ICI)-based regimens, including ICI monotherapy and ICI plus chemotherapy, have become the standard first-line treatment for patients with locally advanced or metastatic LUSC (10–13). PD-1 and PD-L1 inhibitors are the most widely used ICIs in clinical practice. Although PD-L1 expression is routinely applied to predict immunotherapy response, its predictive performance is suboptimal (14, 15). Hence, discovering reliable predictive biomarkers and developing more effective therapeutic strategies has become an urgent priority for LUSC treatment.

LUSC is characterized by a high mutational burden and marked genomic complexity (16). Multiple genes and signaling pathways are frequently dysregulated in this disease, such as TP53, CDKN2A, PTEN, PIK3CA, FGFR1, NOTCH1 and RB1 (17). Among these alterations, copy number (CN) gain of the 8p11–12 chromosomal region harboring the FGFR1 locus is frequently detected in LUSC, occurring in 12%–20% of patients and representing a subtype-enriched genomic alteration (18–20). As a key receptor tyrosine kinase, FGFR1 mediates cell proliferation, differentiation, migration and angiogenesis through the FGF/FGFR signaling cascade (21, 22). Accumulating evidence suggests that aberrant FGFR1 signaling may not only promote tumor cell proliferation and survival but also influence the tumor immune microenvironment (TME) (23–25). Activated FGFR signaling promotes immune evasion by regulating PD-L1 expression, impairing antigen presentation, and suppressing the infiltration and cytotoxic activity of CD8^+^ T cells, as well as other intratumoral immune components (26–28). However, the correlation between FGFR1 CN-gain and immunotherapy response in LUSC remains poorly defined. Prior studies have yielded conflicting results regarding links between this genomic aberration, PD-L1 expression and intratumoral T-cell infiltration. Therefore, it is necessary to further explore whether FGFR1 CN-gain impacts the efficacy of first-line ICI-based therapy for advanced LUSC.

In this retrospective analysis, we enrolled patients with advanced LUSC receiving first-line ICI therapy. Targeted next-generation sequencing was performed to detect FGFR1 CN alterations. Baseline features, treatment response, progression-free survival and overall survival were compared across groups stratified by FGFR1 CN status. Our primary objective was to explore whether FGFR1 CN-gain could serve as a prognostic biomarker for patients receiving first-line ICI treatment.

## 2 Patients and methods

### 2.1 Patients and specimens

In this retrospective cohort study, we enrolled 181 patients with locally advanced or metastatic LUSC who received first-line ICI-based therapy from January 2018 to July 2023 at Shanghai Chest Hospital. The inclusion criteria were as follows: (I) histologically confirmed LUSC staged as IIIB–IV according to the 8th edition of the International Association for the Study of Lung Cancer TNM classification system; (II) available pretreatment next-generation sequencing (NGS) and PD-L1 immunohistochemistry (IHC) results, using formalin-fixed paraffin-embedded or fresh frozen tumor tissue specimens; (III) receipt of first-line ICI-based therapy, including ICI monotherapy or ICI combined with chemotherapy; and (IV) at least one measurable lesion at baseline and available follow-up imaging for radiological response assessment according to Response Evaluation Criteria in Solid Tumors version 1.1 (RECIST 1.1). The exclusion criteria were as follows: absence of NGS or PD-L1 testing, receipt of neoadjuvant therapy, incomplete clinical or molecular data, absence of measurable or reliably evaluable lesions, or lack of follow-up imaging for treatment response assessment.

### 2.2 Clinical assessments

Prior to treatment initiation, all included patients were staged according to the 8th edition of the IASLC TNM classification system. Patients received first-line ICI-based therapy, either as monotherapy or in combination with chemotherapy. During treatment, chest computed tomography (CT)and abdominal ultrasound were performed every 2 to 3 months. Additional cranial magnetic resonance imaging (MRI) and bone scintigraphy were conducted as needed. Assessments continued until disease progression, treatment discontinuation or the final follow-up. Treatment responses were evaluated according to RECIST 1.1 and classified as complete response (CR), partial response (PR), stable disease (SD), or progressive disease (PD). Patients were included in the radiological response analysis only if they had at least one measurable lesion at baseline and available follow-up imaging. For lesions that could not be reliably measured, such as primary lung lesions adjacent to major blood vessels, another measurable lesion meeting RECIST 1.1 criteria, including a metastatic lymph node, was selected as the target lesion when available. The primary endpoint was progression-free survival (PFS), and the secondary endpoint was overall survival (OS),objective response rate (ORR) and disease control rate (DCR). ORR was defined as the proportion of patients with CR or PR, and DCR was defined as the proportion of patients with CR, PR, or SD. The final follow-up date was March 25, 2025.

### 2.3 Targeted next-generation sequencing

DNA was extracted from FFPE tissue using the QIAamp DNA FFPE Tissue Kit. Libraries were prepared following the manufacturer’s protocol and sequenced on the Illumina NextSeq500 platform with paired-end reads and a target depth of 1,000×. Genomic profiling was performed using the Lung Core panel, covering 68 lung cancer-related genes across 245 kb of the genome.

### 2.4 Sequence data analysis

Sequence reads were mapped to the hg19 reference genome using BWA (v0.7.10). Variant calling and annotation were performed using GATK (v3.2) and VarScan (v2). Common germline variants (population frequency >0.1%) were excluded, and remaining variants were annotated with ANNOVAR (v3) and SnpEff (v3.6). DNA translocations were analyzed using standard bioinformatics tools. CN variation was estimated using the bioinformatics pipeline of the testing platform. In the primary analysis, FGFR1 CN alteration was assessed based on the platform-reported threshold. FGFR1 CN status was classified based on the platform-reported threshold: CN >2 as FGFR1 CN-gain, CN ≤2 as non-gain. A sensitivity analysis using CN ≥6 was performed to identify high-level FGFR1 amplification.

### 2.5 Pathological diagnostic and PD-L1 tumor proportion score

Pathological diagnoses were completed by the Department of Pathology at Shanghai Chest Hospital, according to the 5th edition of the WHO classification of thoracic tumors (2021). Diagnoses were reviewed and confirmed by two senior pathologists. All IHC staining procedures and interpretation criteria were conducted according to the manufacturer’s instructions. The expression levels of PD-L1 in tumor cells were assessed using the TPS and categorized as TPS < 1% (negative expression) and TPS ≥1% (positive expression).

### 2.6 Statistical analysis

All statistical analyses were performed using IBM SPSS Statistics (version 26.0), GraphPad Prism (version 9.0), and R (version 4.3.2). Categorical variables among different groups were compared using the chi-square (χ^2^) test or Fisher’s exact test, as appropriate. PFS was calculated from the initiation of first-line ICI-based therapy until disease progression, treatment switch, death, or the date of the last follow-up, whichever occurred first. OS was defined as the time from treatment initiation to death from any cause or the date of the last follow-up. The median follow-up duration was estimated using the reverse Kaplan–Meier method. Kaplan–Meier curves were generated for PFS and OS, and between-group differences were compared using the log-rank test. Univariate and multivariate Cox proportional hazards regression models were used to identify independent prognostic factors. Variables with P < 0.20 in univariate analysis were included in the multivariate model. All statistical tests were two-sided, and a P value < 0.05 was considered statistically significant.

## 3 Result

### 3.1 Patient characteristics

**Figure 1:**
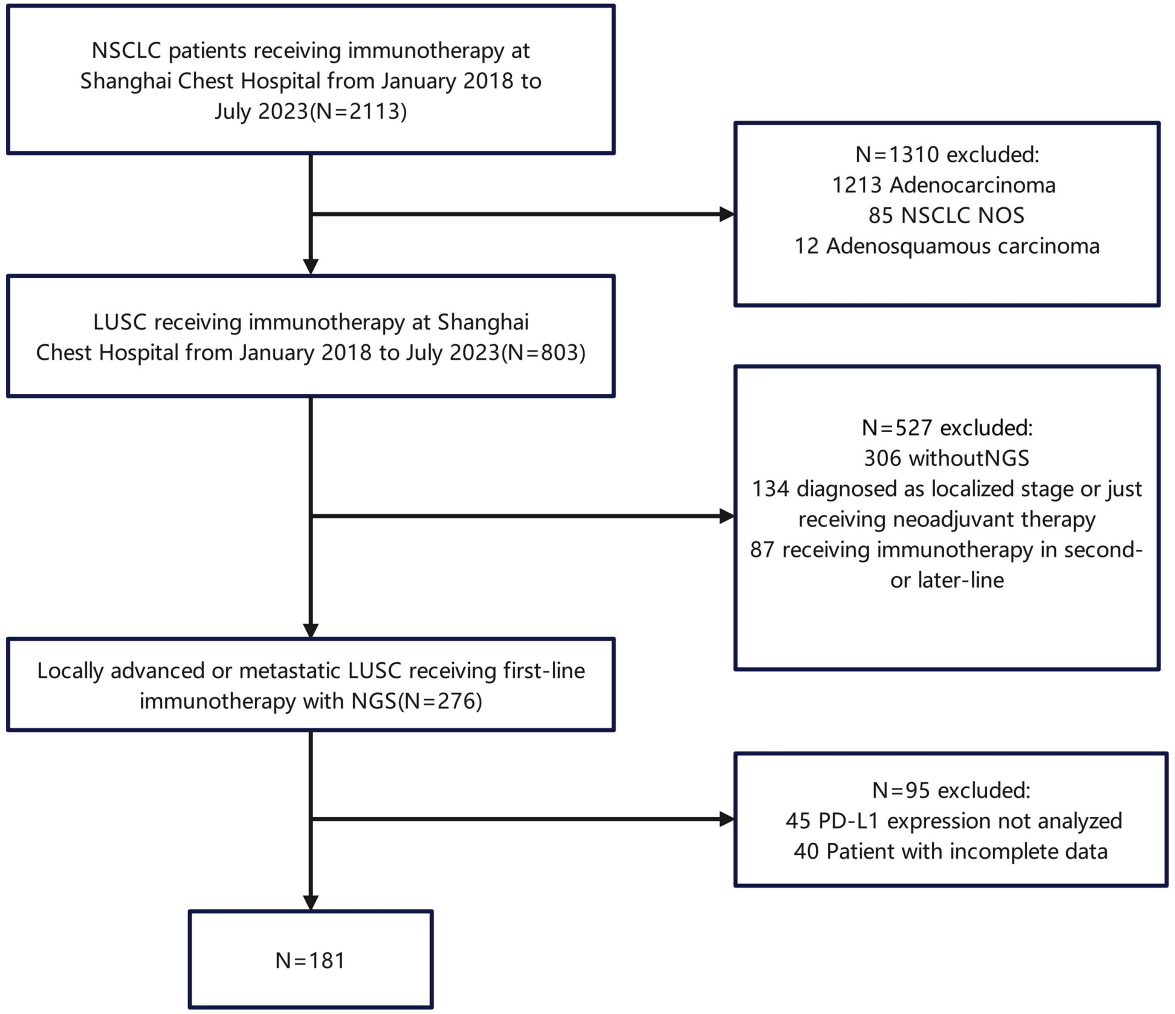
Flowchart of advanced lung squamous cell carcinoma patients receiving first-line immunotherapy.

**Figure 2:**
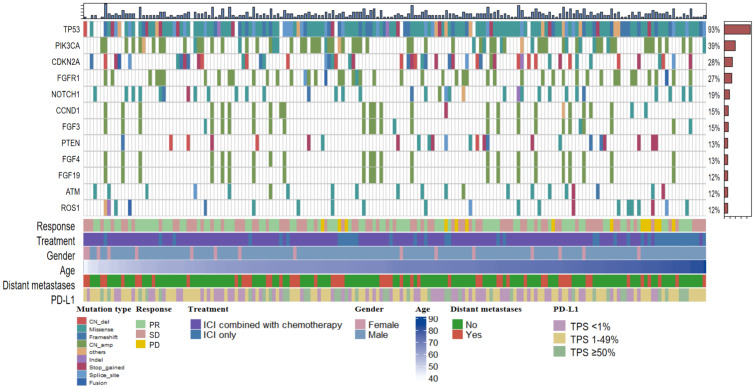
Molecular characteristics of advanced lung squamous cell carcinoma patients receiving first-line immunotherapy.

From January 2018 to July 2023, a total of 2,113 NSCLC patients who received ICI-based therapy were enrolled. After preliminary screening, 181 patients with advanced LUSC who received first-line ICI-based therapy were finally enrolled in this study (**Figure 1**). The mutation frequencies of major genes and patients’ baseline characteristics are summarized below (**Figure 2**). TP53 was the most frequently mutated gene, with an alteration rate of 92.8% (168/181), followed by PIK3CA (38.7%, 70/181), CDKN2A (28.2%, 51/181) and FGFR1(26.5%,48/181). Among the 48 cases with FGFR1 variations, FGFR1 CN-gain was the predominant type (91.7%, 44/48). All participants were stratified into two groups according to FGFR1 CN status: FGFR1 gain (n = 44, 24.3%) and FGFR1 non-gain (n = 137, 75.7%). The median age of the overall cohort was 67 years (range 47–87). The majority of patients were male (n = 165, 91.2%), had a smoking history (n = 148, 81.8%), and presented with an ECOG performance status (PS) ≥ 1 (n = 170, 93.9%). Most patients (71.3%, 129/181) had no distant metastases at baseline. In terms of tumor stage, 85 patients (47%) were at stage III and 96 patients (53%) at stage IV. Regarding the PD-L1 tumor proportion score (TPS), 63 patients (34.8%) had TPS < 1%, 83 patients (45.9%) had TPS 1–49%, and 35 patients (19.3%) had TPS ≥ 50%. First-line regimens consisted of ICI monotherapy (n = 39, 21.5%) and ICI combined with chemotherapy (n = 142, 78.5%). No significant differences in baseline characteristics were detected between the two groups (all P > 0.05) (**Table 1**).

**Table 1:** Characteristics of all patients and patients in different group.

Characteristics	Overall	FGFR1 CN-gain (n=44)(%)	FGFR1 CN-non-gain (n=137)(%)	P
Age (years, M ± SD)	67.4 ± 8.36	68.2 ± 7.41	67.1 ± 8.65	0.548
Smoking				0.175
Yes	148 (81.8%)	39 (21.5%)	109 (60.2%)	
No	33 (18.2%)	5 (2.8%)	28 (15.5%)	
Gender				0.145
Male	165 (91.2%)	43 (23.8%)	122 (67.4%)	
Female	16 (8.8%)	1 (0.6%)	15 (8.3%)	
PD-L1 TPS				0.587
TPS<1%	63 (34.8%)	18 (9.9%)	45 (24.9%)	
TPS 1-49%	83 (45.9%)	19 (10.5%)	64 (35.4%)	
TPS≥50%	35 (19.3%)	7 (3.9%)	28 (15.5%)	
ECOG PS score				0.706
0	11 (6.1%)	3 (1.7%)	8 (4.4%)	
1	168 (92.8%)	41 (22.7%)	127 (70.2%)	
2	2 (1.1%)	0 (0%)	2 (1.1%)	
Clinical Stage				0.907
III	85 (47%)	21 (11.6%)	64 (35.4%)	
IV	96 (53%)	23 (12.7%)	73 (40.3%)	
Distant metastasis				0.530
Yes	52 (28.7%)	11 (6.1%)	41 (22.7%)	
No	129 (71.3%)	33 (18.2%)	96 (53%)	
First-line treatment				0.827
ICI only	39 (21.5%)	10 (5.5%)	29 (16%)	
ICI combined with chemotherapy	142 (78.5%)	34 (18.8%)	108 (59.7%)	

### 3.2 Treatment response and survival outcomes according to FGFR1 CN status

Among all 181 patients, 0 achieved complete response (CR), 89 (49.2%) achieved partial response (PR), 77 (42.5%) had stable disease (SD), and 15 (8.3%) experienced progressive disease (PD), yielding an overall disease control rate (DCR) of 91.7% (**Table 2**). Stratified by FGFR1 CN status, the distribution of responses differed significantly (P = 0.023). In the CN-gain group, 20 (45.4%) achieved PR, 16 (36.4%) had SD, and 8 (18.2%) experienced PD, compared with 69 (50.4%), 61 (44.5%), and 7 (5.1%) in the CN-non-gain group, respectively. The ORR (CR+PR) was similar between groups (45.4% vs. 50.4%, P = 0.571), whereas the DCR was lower in the CN-gain group (81.8% vs. 94.9%, P = 0.015).

**Table 2:** Treatment response and survival outcomes in patients with advanced LUSC according to FGFR1 CN status.

Efficacy	Total n=181	FGFR1 CN-gain n=44	FGFR1 CN-non-gain n=137	P
CR	0	0	0	0.023
PR	89 (49.2%)	20 (45.4%)	69 (50.4%)	
SD	77 (42.5%)	16 (36.4%)	61 (44.5%)	
PD	15 (8.3%)	8 (18.2%)	7 (5.1%)	
ORR	49.2%	45.4%	50.4%	0.571
DCR	91.7%	81.8%	94.9%	0.015
Median PFS, months (95% CI)	9.0(7.5-10.5)	7.7(5.4-10.1)	9.5(7.1-11.9)	0.025
Median OS, months (95% CI)	20.1(15.3-24.9)	18.8(13.0-24.6)	22.8(17.5-28.1)	0.448

*Categorical variables (including ORR, DCR, and best response categories) were compared using Pearson’s χ² test, with Fisher’s exact test applied when the expected frequency in any cell was less than 5.

**Figure 3:**
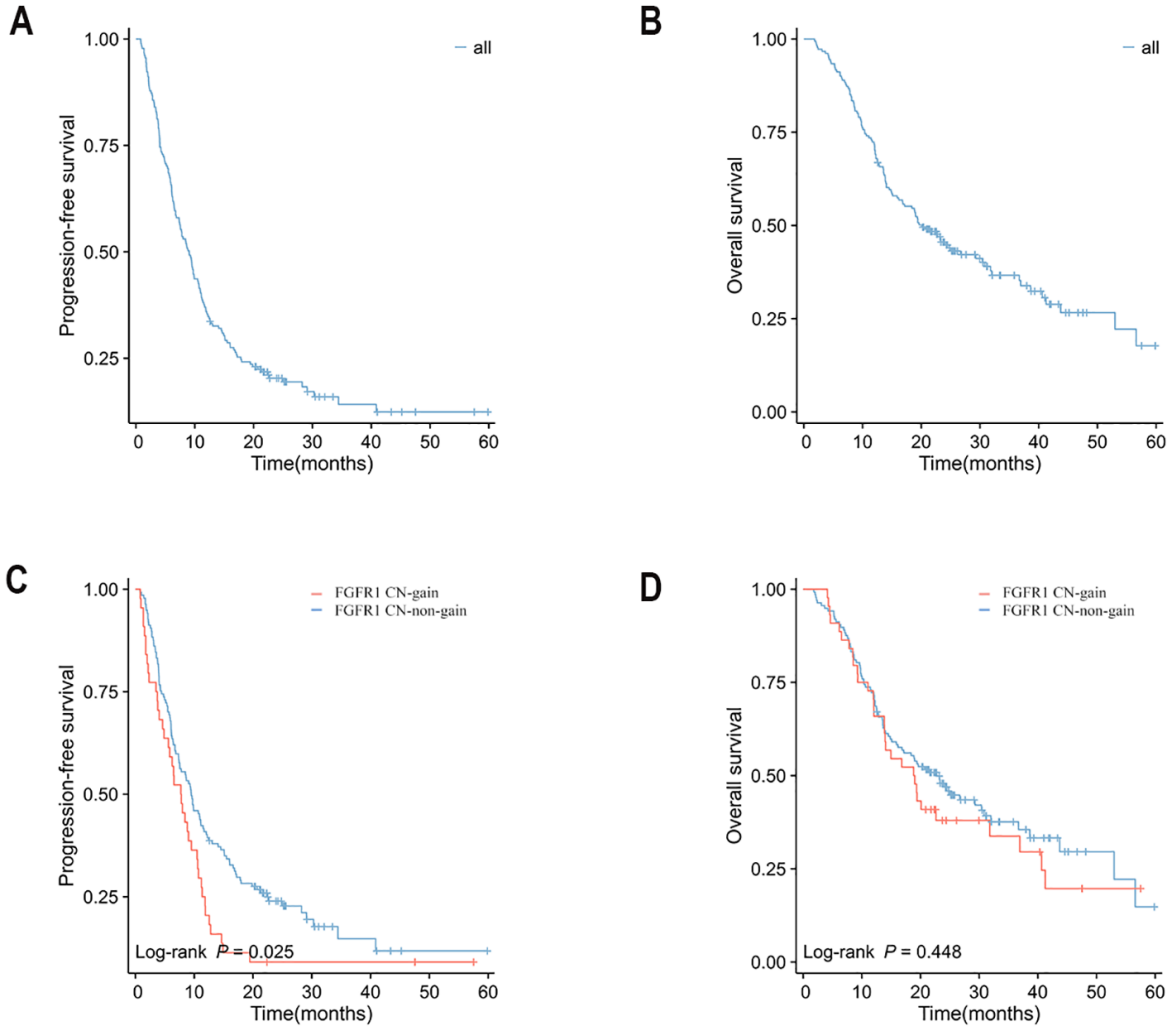
Kaplan–Meier curves of survival outcomes according to FGFR1 CN status. **(A)** Progression-free survival for all patients. **(B)** Overall survival for all patients. **(C)** Survival curve of progression-free survival in FGFR1CN-gain vs. CN-non-gain groups. **(D)** Survival curve of overall survival in FGFR1 CN-gain vs. CN-non-gain groups.

The median follow-up duration was 31.2 months (95% CI: 26.8–35.5). For all enrolled patients, the median PFS was 9.0 months (95% CI: 7.5–10.5), and the median OS was 20.1 months (95% CI: 15.3–24.9). Patients with FGFR1 CN-gain had significantly shorter PFS than those without CN-gain (7.7 months,95% CI: 5.4–10.1 vs. 9.5 months, 95% CI: 7.1–11.9, P = 0.025). OS did not differ significantly (18.8 months, 95% CI: 13.0–24.6 vs. 22.8 months, 95% CI: 17.5–28.1, P = 0.448) (**Figure 3**).

### 3.3 Cox regression analysis for PFS and OS

Univariate Cox regression analysis showed that FGFR1 CN-gain was associated with shortened PFS in patients (HR = 1.513, 95% CI: 1.050–2.180, P = 0.026). Clinical stage showed a trend toward association with PFS (stage IV vs. stage III: HR = 1.296, 95% CI: 0.935–1.796, P = 0.120). In the multivariate Cox regression model, FGFR1 CN-gain remained independently associated with shorter PFS (HR = 1.554, 95% CI: 1.077–2.242, P = 0.018), whereas clinical stage did not reach statistical significance (HR = 1.334, 95% CI: 0.962–1.851, P = 0.084) (**Table 3**).

**Table 3:** Univariable and multivariable analysis for progression-free survival (PFS) in all patients.

Characteristics	Univariate analysis		Multivariate analysis	
	Hazard ratio (95% CI)	P value	Hazard ratio (95% CI)	P value
Age				
<65 years	Reference			
≥65 years	1.216(0.850-1.738)	0.284		
Gender				
Male	Reference			
Female	0.933 (0.517 - 1.685)	0.818		
Smoking				
No	Reference			
Yes	1.073(0.702-1.639)	0.746		
Clinical stage
III	Reference		Reference	
IV	1.296 (0.935 - 1.796)	0.120	1.334 (0.962 - 1.851)	0.084
Distant metastases				
No	Reference			
Yes	1.183 (0.831 - 1.684)	0.350		
PD-L1 TPS				
TPS<1%	Reference			
TPS 1-49%	0.799 (0.559 - 1.143)	0.220		
TPS≥50%	0.728 (0.461 - 1.149)	0.173		
First-line treatment				
ICI only	Reference			
ICI combined with chemotherapy	1.061 (0.713 - 1.577)	0.770		
PIK3CA status				
CN-non-gain	Reference			
CN-gain	0.979 (0.690 - 1.389)	0.903		
FGFR1 status				
CN-non-gain	Reference		Reference	
CN-gain	1.513 (1.050 - 2.180)	**0.026**	1.554 (1.077 - 2.242)	**0.018**

Bold values denote statistically significant P < 0.05. CI = confidence interval; CN = copy number; ICI = immune checkpoint inhibitor; TPS = tumor proportion score.

For OS, FGFR1 CN-gain was not significantly associated with survival in univariate analysis (HR = 1.173, 95% CI: 0.776–1.774, P = 0.448). Smoking status and clinical stage showed nonsignificant trends and were included in the multivariate model, but neither remained significantly associated with OS (smoking: HR = 1.521, 95% CI: 0.916–2.528, P = 0.105; stage IV vs. stage III: HR = 1.343, 95% CI: 0.919–1.963, P = 0.128) (**Table 4**).

**Table 4:** Univariable and multivariable cox regression analysis for overall survival (OS) in all patients.

Characteristics	Univariate analysis		Multivariate analysis	
	Hazard ratio (95% CI)	P value	Hazard ratio (95% CI)	P value
Age				
<65 years	Reference			
≥65 years	1.107 (0.736 - 1.664)	0.625		
Gender				
Male	Reference			
Female	0.789 (0.384 - 1.620)	0.518		
Smoking				
No	Reference		Reference	
Yes	1.502 (0.904 - 2.496)	0.117	1.521 (0.916 - 2.528)	0.105
Clinical stage				
III	Reference		Reference	
IV	1.326 (0.908 - 1.937)	0.144	1.343 (0.919 - 1.963)	0.128
Distant metastases				
No	Reference			
Yes	1.235 (0.829 - 1.838)	0.299		
PD-L1 TPS				
TPS<1%	Reference			
TPS 1-49%	0.882 (0.584 - 1.331)	0.550		
TPS≥50%	0.833 (0.502 - 1.383)	0.479		
First-line treatment				
ICI only	Reference			
ICI combined with chemotherapy	0.876 (0.569 - 1.351)	0.550		
PIK3CA status				
CN-non-gain	Reference			
CN-gain	1.036 (0.697 - 1.538)	0.863		
FGFR1 status				
CN-non-gain	Reference			
CN-gain	1.173 (0.776 - 1.774)	0.448		

### 3.4 Subgroup and sensitivity analyses

Among the 142 patients receiving first-line ICI combined with chemotherapy, 34 were classified as FGFR1 CN-gain and 108 as CN-non-gain. The ORR was comparable between the two groups (52.9% vs. 53.7%, P = 0.938), while the DCR was numerically lower in the CN-gain group (88.2% vs. 97.2%, P = 0.098) (**Supplementary Table 1**). Kaplan–Meier analysis revealed that patients with FGFR1 CN-gain had significantly shorter PFS compared with those without CN-gain (7.8 months, 95% CI: 5.1–10.5 vs. 9.5 months,95% CI: 7.7–11.2, P = 0.009). In contrast, OS was not significantly different between the groups (16.8 months, 95% CI: 9.3–24.3 vs. 25.1 months, 95% CI: 15.7–34.4, P = 0.310) (**Supplementary Figure 1**).

A sensitivity analysis was performed using FGFR1 CN ≥ 6 to define high-level amplification. Among 181 patients, 16 were classified as high-level CN (CN ≥ 6) and 165 as CN < 6 (**Supplementary Table 2**). Kaplan–Meier analysis showed a trend toward shorter PFS in patients with high-level CN compared with those with CN <6 (P = 0.105). OS was also not significantly different between the groups (P = 0.087) (**Supplementary Figure 2**).

## 4 Discussion

In this retrospective cohort study of 181 patients with advanced LUSC receiving first-line ICI-based therapy, we found that FGFR1 CN-gain was associated with inferior disease control and significantly shorter PFS (29, 30). Although ORR was comparable between the FGFR1 CN-gain and CN-non-gain groups, patients with FGFR1 CN-gain had a significantly lower DCR and a higher proportion of early disease progression. Multivariate Cox regression further confirmed that FGFR1 CN-gain was independently associated with shorter PFS after adjustment for relevant clinicopathological factors. In contrast, FGFR1 CN-gain was not significantly associated with OS. These findings suggest that FGFR1 CN-gain may be more closely related to the durability of disease control rather than the initial radiological response to first-line ICI-based therapy.

The distinction between ORR and DCR is clinically relevant (31, 32). In our cohort, FGFR1 CN-gain did not significantly reduce the probability of achieving PR, but it was associated with a higher proportion of PD and a lower DCR. This pattern suggests that FGFR1 CN-gain may not primarily impair early tumor shrinkage, particularly in patients receiving combination treatment, but may contribute to earlier treatment failure or reduced maintenance of disease control. Therefore, PFS may better capture the adverse clinical impact of FGFR1 CN-gain than ORR alone. This is also consistent with the multivariate analysis, in which FGFR1 CN-gain remained an independent factor associated with shorter PFS.

Because our cohort included both ICI monotherapy and ICI combined with chemotherapy, we further performed a subgroup analysis restricted to patients receiving chemo-immunotherapy. This analysis is clinically important because ICI plus chemotherapy represents a widely used first-line treatment strategy for advanced LUSC (31, 33). In this subgroup, ORR remained comparable between the FGFR1 CN-gain and CN-non-gain groups, while DCR was numerically lower in the FGFR1 CN-gain group. More importantly, FGFR1 CN-gain remained significantly associated with shorter PFS, whereas OS was not significantly different. These findings indicate that the adverse association between FGFR1 CN-gain and PFS was not solely driven by patients treated with ICI monotherapy. They also suggest that chemotherapy may not fully overcome the negative impact of FGFR1 CN-gain on the durability of clinical benefit from ICI-based treatment.

We also conducted a sensitivity analysis using FGFR1 CN ≥6 as the cutoff for high-level amplification (34). This analysis was designed to address the potential influence of different CN thresholds, as CN >2 may include low-level CN-gain, whereas CN ≥6 more closely reflects high-level amplification. In this exploratory analysis, patients with high-level FGFR1 amplification showed a trend toward shorter PFS compared with those with FGFR1 CN <6, although the difference did not reach statistical significance. OS was also not significantly different between the two groups. The lack of statistical significance may be partly attributable to the small number of patients with high-level amplification. Nevertheless, the direction of the effect was consistent with the primary analysis, supporting the possibility that higher FGFR1 CN may be associated with reduced benefit from first-line ICI-based therapy.

The relationship between FGFR1 alteration and the tumor immune microenvironment may provide a biological explanation for our findings. Aberrant activation of the FGF/FGFR pathway has been implicated in tumor proliferation, angiogenesis, immune evasion, and resistance to anticancer therapy (35). FGFR signaling may influence antitumor immunity through several mechanisms, including impaired antigen presentation, reduced infiltration or function of cytotoxic T cells, recruitment of immunosuppressive myeloid cells, and modulation of PD-L1 expression (27). These changes could weaken durable immune-mediated tumor control and contribute to earlier disease progression during ICI-based therapy (36). However, immune cell infiltration, antigen-presentation markers, cytokine profiles, and spatial features of the tumor microenvironment were not directly assessed in the present study. Therefore, these mechanistic interpretations remain hypothesis-generating and require further validation in translational studies.

PD-L1 expression remains the most widely used biomarker for selecting patients for ICI-based therapy, but its predictive performance is imperfect (37–39). In response to this issue, we reclassified PD-L1 TPS into clinically relevant categories of <1%, 1–49%, and ≥50%. After incorporating this three-category PD-L1 classification into the Cox regression models, FGFR1 CN-gain remained independently associated with shorter PFS. In addition, the distribution of PD-L1 TPS categories did not differ significantly between the FGFR1 CN-gain and CN-non-gain groups. These findings suggest that the adverse association between FGFR1 CN-gain and PFS was unlikely to be explained simply by enrichment of PD-L1–low tumors in the FGFR1 CN-gain group. Instead, FGFR1 CN-gain may represent a distinct molecular feature associated with reduced durability of benefit from ICI-based treatment.

The absence of a significant OS difference deserves careful interpretation. PFS more directly reflects the efficacy of first-line ICI-based therapy, whereas OS can be influenced by multiple post-progression factors, including later-line chemotherapy, antiangiogenic therapy, local treatment, treatment switching, supportive care, and the patient’s condition after progression. In our cohort, some later-line treatment information was available, but these data were incomplete and were not systematically captured for all patients. Therefore, subsequent therapies were not included in the multivariate OS model, as adjustment based on incomplete treatment-sequence data could introduce additional bias. The limited number of OS events and the retrospective nature of the study may also have reduced the statistical power to detect an OS difference. Thus, the lack of a statistically significant OS association does not necessarily exclude the clinical relevance of FGFR1 CN-gain.

From a clinical perspective, FGFR1 CN-gain may help identify patients with advanced LUSC who are at higher risk of early progression during first-line ICI-based therapy. This may be particularly relevant for patients receiving chemo-immunotherapy, in whom early response rates may appear similar despite differences in PFS. If validated prospectively, FGFR1 CN-gain could be incorporated into molecular risk stratification models together with established clinical and immunological markers, such as PD-L1 TPS and disease burden (40, 41). In addition, our findings provide a rationale for future studies exploring combination strategies involving FGFR pathway inhibition and immune checkpoint blockade.

Several limitations should be acknowledged. First, this was a retrospective single-center study with inherent selection bias. The relatively limited sample size, particularly in the ICI plus chemotherapy subgroup and patients with high-level FGFR1 amplification (CN ≥6), may constrain the generalizability of our findings. Second, although treatment regimens were adjusted for in the multivariate analysis, residual treatment heterogeneity and incomplete data on subsequent therapies may compromise the assessment of long-term survival outcomes. In addition, this study lacked comprehensive profiling of the tumor immune microenvironment and tumor mutational burden (TMB). Given the critical role of TMB and immune landscape in modulating tumor immunogenicity and ICI response, the lack of these data prevented us from fully characterizing the intrinsic immune-molecular features of FGFR1-altered LUSC and ruling out confounding factors for immunotherapy efficacy (20, 42). Finally, FGFR1 CN was determined via targeted NGS, and relevant cut-off values may differ across detection platforms and bioinformatics pipelines. Accordingly, large-scale prospective multicenter studies with standardized testing protocols and combined molecular and immune analyses are warranted.

In conclusion, FGFR1 CN-gain identifies patients with advanced LUSC who are at higher risk of early progression during first-line ICI-based therapy, particularly in those receiving chemo-immunotherapy. While its impact on ORR and OS was not significant, FGFR1 CN-gain may serve as a potential biomarker to guide risk stratification and treatment planning. Prospective multicenter studies integrating standardized molecular profiling, treatment-sequence information, and tumor immune microenvironment analyses are warranted to validate these findings and to explore FGFR-targeted combination strategies.

## Data Availability

The raw clinical and FGFR1 NGS datasets contain human genetic information. Restricted by national human genetic resource regulations and Shanghai Chest Hospital privacy policies, these raw data cannot be deposited in public repositories at home or abroad. All anonymized aggregate data supporting the study findings are included in the manuscript and supplementary materials. Eligible researchers may request de-identified raw data from the corresponding author after institutional ethics approval.
