# Supplementary material for: FGFR1 copy number gain is independently associated with shorter progression-free survival in advanced lung squamous cell carcinoma treated with first-line immune checkpoint inhibitor-based therapy
Source: Front Immunol. 2026 Jul 29;17:1718660. doi: 10.3389/fimmu.2026.1718660 (PMC13462056; doi:10.3389/fimmu.2026.1718660)
Supplement: Supplementary file 1 [file Table1.docx]

Figure S1. Association of FGFR1 copy number gain with survival in the chemo-immunotherapy cohort.

| **Efficacy** | **Total**  **n=142** | **FGFR1 CN-gain**  **n=34** | **FGFR1 CN-non-gain**  **n=108** | **P** |
| --- | --- | --- | --- | --- |
| CR | 0 | 0 | 0 |  |
| PR | 76 (53.5%) | 18 (52.9%) | 58 (53.7%) |  |
| SD | 59 (41.5%) | 12 (35.3%) | 47 (43.5%) |  |
| PD | 7 (4.9%) | 4 (11.8%) | 3 (2.8%) |  |
| ORR | 53.5% | 52.9% | 53.7% | 0.938 |
| DCR | 95.1% | 88.2% | 97.2% | 0.098 |
| Median PFS, months (95% CI) | 9.0（7.7-10.4） | 7.8（5.1-10.5） | 9.5（7.7-11.2） | 0.009 |
| Median OS, months (95% CI) | 19.6(12.2-27.0) | 16.8(9.3-24.30 | 25.1(15.7-34.4) | 0.310 |

Table S1.Treatment response and survival outcomes in patients receiving first-line ICI combined with chemotherapy stratified by FGFR1 copy number status.

Categorical variables (including ORR, DCR, and best response categories) were compared using Pearson’s χ² test, with Fisher’s exact test applied when the expected frequency in any cell was less than 5. Kaplan-Meier survival curves were plotted for PFS and OS, and between-group differences were assessed using the log-rank test.

Figure S2. Association of high FGFR1 copy number with survival across all immunotherapy-treated patients.

| **Efficacy** | **Total**  **n=181** | **FGFR1 CN ≥6**  **n=16** | **FGFR1 CN <6**  **n=165** | **P** |
| --- | --- | --- | --- | --- |
| CR | 0 | 0 | 0 |  |
| PR | 89 (49.2%) | 8(50.0%) | 81 (49.1%) |  |
| SD | 77(42.5%) | 5(31.3%) | 72 (43.6%) |  |
| PD | 15(8.3%) | 3(18.8%) | 12 (7.3%) |  |
| ORR | 49.2% | 50.0% | 49.1% | 0.94 |
| DCR | 91.7% | 81.3% | 92.7% | 0.001 |
| Median PFS, months (95% CI) | 9.0（7.5-10.5） | 7.8（5.0-10.6） | 9.4（7.7-11.1） | 0.105 |
| Median OS, months (95% CI) | 20.1（15.3-24.9） | 12.1（8.4-15.7） | 22.8（17.3-28.3） | 0.087 |

Table S2.Treatment response and survival outcomes according to high-level FGFR1 copy number (CN ≥6).

Categorical variables (including ORR, DCR, and best response categories) were compared using Pearson’s χ² test, with Fisher’s exact test applied when the expected frequency in any cell was less than 5. Kaplan-Meier survival curves were plotted for PFS and OS, and between-group differences were assessed using the log-rank test.
